# Mechanisms underlying the promotion of papillary thyroid carcinoma occurrence and progression by Hashimoto’s thyroiditis

**DOI:** 10.3389/fendo.2025.1551271

**Published:** 2025-03-31

**Authors:** Xiaohui Xue, Deqi Wu, Hangyu Yao, Kainan Wang, Zhengtao Liu, Haijiang Qu

**Affiliations:** ^1^ School of Medicine, Zhejiang Chinese Medical University, Hangzhou, China; ^2^ Department of Thyroid and Breast Diagnosis and Treatment Center, Shulan (Hangzhou) Hospital, Shulan International Medical College, Zhejiang Shuren University, Hangzhou, China; ^3^ Key Laboratory of Artificial Organs and Computational Medicine in Zhejiang Province, Shulan International Medical College, Zhejiang Shuren University, Hangzhou, China; ^4^ Department of Hepatobiliary Surgery, Shulan (Hangzhou) Hospital, Shulan International Medical College, Zhejiang Shuren University, Hangzhou, China; ^5^ NHC Key Laboratory of Combined Multi-Organ Transplantation, Key Laboratory of the Diagnosis and Treatment of Organ Transplantation, First Affiliated Hospital, School of Medicine, Zhejiang University, Hangzhou, China; ^6^ Key Laboratory of Organ Transplantation, First Affiliated Hospital, School of Medicine, Zhejiang University, Hangzhou, China

**Keywords:** Hashimoto’s thyroiditis, autoimmune thyroid diseases, papillary thyroid cancer, microenvironment, regulatory T cells, signaling pathways

## Abstract

Hashimoto’s thyroiditis (HT) and papillary thyroid carcinoma (PTC) co-occurrence raises significant questions regarding the immune microenvironment and molecular mechanisms in thyroid tumor development. This review synthesizes recent literature to explore the immune microenvironment and molecular characteristics of PTC patients with HT, and to analyze how these characteristics influence disease onset, progression, and treatment. We focused on the immunological and molecular biological mechanisms underlying the interaction between HT and PTC, particularly the recruitment and activation of immune cells and alterations in key signaling pathways. Studies indicate that PTC with HT exhibits distinctive immune microenvironmental features, such as the role of regulatory T cells (Tregs), activation of the IFN-γ-mediated CXCR3A-CXCL10 signaling axis, and NF-κB pathway activation. Additionally, thyroid-stimulating hormone (TSH) stimulation, *RET/PTC* gene rearrangements, and changes in STAT6 and *DMBT1* gene expression levels also play significant roles in PTC development. Notably, while HT may increase the risk of PTC, patients with concurrent HT tend to have better prognoses. Future research should further elucidate the complex interplay between these two diseases to prevent the transformation of HT into PTC and offer more personalized treatment plans for PTC patients, including considerations for preoperative thyroidectomy and lymph node dissection strategies, as well as postoperative TSH suppression therapy risk assessment. This review underscores the importance of a deeper understanding of HT and PTC interactions and offers new perspectives for future research directions and therapeutic strategies.

## 1 Introduction

Autoimmune Thyroid Diseases (AITDs) result from pathological activation of the immune system against the thyroid gland (1),characterized primarily by lymphocytic infiltration of the thyroid parenchyma. The main conditions included are Graves’ Disease (GD) and Hashimoto’s Thyroiditis (HT).

HT, also known as chronic lymphocytic thyroiditis or autoimmune thyroiditis, is characterized by thyroid enlargement, follicular hyperplasia, and infiltration of plasma cells and lymphocytes (2). It is an autoimmune disease defined by the cell-and antibody-mediated destruction of thyroid cells (3). In the microenvironment of the thyroid gland in HT patients, there is a significant presence of infiltrating lymphocytes and other immunoactive cells, along with various soluble mediators such as chemokines, cytokines, and growth factors, all of which contribute to the disease’s onset and progression. The incidence of HT has been on the rise in recent years (4). A systematic review and meta-analysis by Hu et al. found a global prevalence of 7.5% in adults with HT, with adult women having approximately four times the prevalence of men, and noted variations in prevalence across regions with different economic statuses (2). Moreover, HT is closely associated with various autoimmune diseases, such as diabetes and rheumatoid arthritis (5). Although the precise etiology of HT is not fully understood, it is believed to be closely associated with genetic influences, environmental triggers, and epigenetic effects, with T-cell infiltration in the thyroid being one of the key factors (6, 7). Thus, further investigation into these potential mechanisms is crucial for understanding the pathophysiology of HT and for developing novel therapeutic strategies.

Thyroid carcinoma (TC) is the most prevalent malignant disease of the endocrine system, with papillary thyroid cancer (PTC) accounting for over 90% of all thyroid cancers. The incidence of TC is rising globally, posing a growing public health challenge and placing a substantial burden on healthcare systems and societal resources (8, 9). To date, ionizing radiation is the only etiology scientifically proven to be associated with the development of TC (10).

In 1955, Dailey first examined the relationship between HT and PTC (11). Subsequent epidemiological studies have revealed a strong coexistence between HT and PTC. Epidemiological reports estimate an average comorbidity rate of 23% (ranging from 5% to 85%) between HT and PTC (12, 13). A recent study including 9,210 patients found a 19% incidence of HT in conjunction with PTC (14). Moreover, a meta-analysis of 10,648 PTC cases indicates that HT is more prevalent in PTC than in benign thyroid diseases and other cancers (13).

The association between inflammation and cancer is widely acknowledged, dating back to Virchow’s 1863 observation of leukocytes in tumor tissues and his hypothesis that they might be related to tumor development (15, 16). Over time, the scientific community has progressively uncovered the role of inflammation in tumor progression. Specifically, studies have confirmed that in certain tissues, the persistent stimulation of chronic inflammation can activate gene expression associated with the inflammatory response, thereby triggering oncogenic signaling pathways and leading to the formation of precancerous lesions (17). In this process, various bioactive molecules released by inflammatory cells, including cytokines, chemokines, and growth factors, play a crucial role (18). These molecules promote cell proliferation, inhibit apoptosis, and foster angiogenesis, collectively creating a supportive microenvironment for tumor growth and progression. In summary, there is a close link between HT and PTC.

Studies have identified HT as an independent risk factor for the development of PTC (19). Patients with PTC and concurrent HT (PTC+HT) exhibit a higher rate of multifocality (19, 20), suggesting that HT may contribute to the onset and progression of PTC. However, numerous studies indicate that compared to patients with PTC alone, those with PTC and HT have fewer lymph node metastases (LNM), extrathyroidal extension, recurrence rates, and mortality, as well as smaller tumor sizes and longer disease-free survival periods (19, 21–24), suggesting a potential protective role of HT in thyroid cancer. It has been reported that the BRAF^V600E^ mutation is a risk factor for LNM in PTC patients and may also be a risk factor for recurrence (23, 25). However, studies show that HT is significantly less common in the BRAF^V600E^-mutated PTC group (20). Moreover, in patients with BRAF wild-type Differentiated Thyroid Carcinoma (DTC), HT is an independent protective factor that can reduce the risk of recurrence by 70% (23). In summary, some studies suggest that the presence of HT is associated with less aggressive PTC.

However, to date, there is no definitive evidence to confirm whether HT promotes the development of PTC and/or plays a protective role in the progression of PTC.This review aims to systematically review and analyze the existing literature to explore the potential mechanisms by which HT may promote the occurrence and development of PTC. Our goal is to deeply analyze the impact of HT on the biological behavior of PTC. Clarifying this interaction is crucial for understanding the relationship between HT and PTC and significant for guiding the development of more precise clinical treatment strategies, preventing HT-associated PTC, and optimizing patient outcomes.

## 2 Immune cells and molecules associated with HT

It is well known that T cells can be categorized into several distinct subtypes, such as CD4+ T cells (Helper T cells), CD8+ T cells (Cytotoxic T lymphocytes, CTLs), regulatory T cells (Tregs), and other subtypes. Studies have demonstrated a significant increase in the infiltration of CD4+ T cells in patients with HT (14). The sensitization of autoreactive CD4+ T cells to thyroid antigens appears to be the initial event in the disease’s pathogenesis (16). Based on differences in surface molecules and intracellular cytokines, CD4+ T cells can be further differentiated into various subsets, such as Th1, Th2, Th9, Th17, Th22, and Tregs.

**Figure 1:**
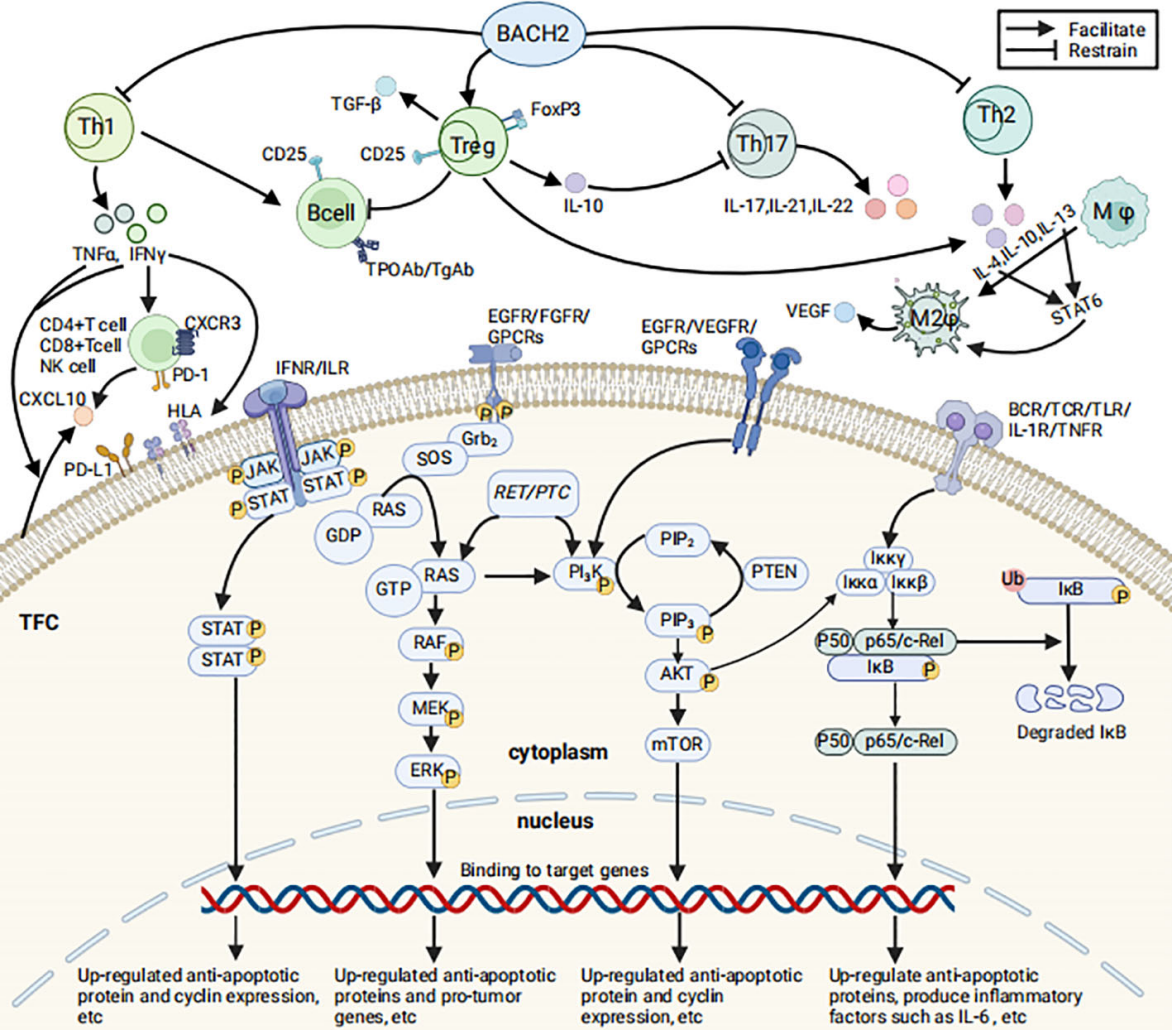
Immune cells/molecules associated with HT and the related signal transduction pathways (Created in BioRender. HUI, X. (2025) https://BioRender.com/d99g053). The figure illustrates selected immune cells and immune molecules associated with HT, their mechanisms of production and functions, as well as the JAK/STAT, MAPK, PI_3_K/AKT, and NF-κB signaling pathways that may contribute to the development and progression of PTC related to HT. The relevant signaling pathways are described as follows: 1) NF-κB pathway: TNF-α and IL-1β activate the IκB kinase (IKK) complex, leading to the phosphorylation, ubiquitination, and subsequent proteasomal degradation of IκB proteins. Following IκB degradation, NF-κB dimers (such as p65/p50) are released from the cytoplasm and translocate to the nucleus, upregulating the expression of anti-apoptotic proteins (e.g., c-IAP1, c-IAP2, XIAP, Bcl-xL), inflammatory cytokines (e.g., IL-6, IL-8), and cell cycle proteins (e.g., Cyclin D1). These changes promote cell survival, proliferation, and inflammatory responses (105). 2) JAK/STAT pathway: IL-6 and IFN-γ activate JAK kinases upon binding to their receptors. Activated JAKs further phosphorylate STAT proteins, promoting their dimerization and nuclear translocation to regulate the transcription of target genes. This process significantly upregulates the expression of anti-apoptotic proteins (e.g., Bcl-2 family members), immune-related genes (e.g., *IL-2Rγ, IRF1*), and cell cycle proteins (e.g., Cyclin D1), thereby promoting cell proliferation, inhibiting apoptosis, and enhancing immune cell infiltration, which drives the development and progression of PTC (106). 3) MAPK pathway: The MAPK pathway can be activated through multiple mechanisms: binding of ligands to receptor tyrosine kinases such as EGFR and FGFR activates RAS via the Grb2-SOS pathway (107, 108); CXCL10-CXCR3A activates RAS through the Gαi subunit (70); *RET/PTC* rearrangements activate the RET kinase domain, which in turn activates RAS (80). Activated RAS interacts with RAF, which phosphorylates MEK, leading to the activation of ERK1/2 and the regulation of nuclear transcription factors. This process upregulates cell cycle proteins (e.g., Cyclin D1) and oncogenes (e.g., *VEGFA, MET*) while downregulating tumor suppressor genes (e.g., *TIMP3, SLC5A8*), thereby regulating cell proliferation, migration, and survival and driving the malignant progression of PTC cells (107, 108). 4) PI_3_K/AKT pathway: Receptor tyrosine kinases (RTKs) such as EGFR and VEGFR auto-phosphorylate upon ligand binding, activating PI_3_K. G protein-coupled receptors (GPCRs) can also activate PI_3_K through different mechanisms. For example, CXCL10 binding to CXCR3 indirectly activates PI_3_K via the Gαq subunit (70). The RET tyrosine kinase activated by *RET/PTC* rearrangements can directly bind to the regulatory subunit of PI_3_K, activating it. PI_3_K phosphorylates PIP_2_ to PIP_3_, which activates AKT. AKT promotes the expression of anti-apoptotic genes (e.g., *Bcl-2, Bcl-x*L) and reduces the expression of cell cycle inhibitors, driving cell proliferation, survival, and invasion, inhibiting apoptosis, and promoting cancer development (109). 5) In summary, the NF-κB and JAK/STAT pathways synergistically promote cell survival and proliferation by upregulating anti-apoptotic proteins (e.g., Bcl-2, Bcl-xL) and cell cycle proteins (e.g., Cyclin D1). NF-κB activation induces the expression and secretion of IL-6, which further amplifies the pro-tumorigenic effects of NF-κB through the JAK/STAT pathway. Additionally, RAS activation enhances the expression of anti-apoptotic proteins via the PI_3_K/AKT pathway, thereby exerting pro-tumorigenic effects. Activated AKT can also phosphorylate the IKK complex, promoting IκB degradation and activating the NF-κB pathway. These signaling pathways collectively drive the proliferation, survival, and invasion of PTC cells through cross-activation and positive feedback mechanisms. (1) TFC, Thyroid Follicular Cells; (2) BACH2, A key protein regulating the immune response; (3) CD4+T cell, A subpopulation of T cells that express CD4 molecules on their surface; (4) CD8+T cell, A subpopulation of T cells that express CD8 molecules on their surface; (5) Th, T helper cell; (6) Treg, Regulatory T cells; (7) FoxP3, A member of the forkhead transcription factor family; (8) B cell, B-lymphocytel; (9) NK cell, Natural killer cell; (10) CD25, The alpha chain of the interleukin-2 receptor (IL-2Rα); (11) TPOAb, Thyroid Peroxidase Antibodies; (12) TgAb, Thyroglobulin Antibodies; (13) Mφ, Macrophage; (14) M2φ, M2Macrophage; (15) IL, Interleukin; (16) IL-1R, Interleukin-1 receptor; (17) INF-γ, Interferon-γ; (18) TNF-α, Tumor Necrosis Factor-α; (19) TNFR, Tumor Necrosis Factor receptor; (20) TGF-β, Transforming Growth Factor-β; (21) VEGF, Vascular Endothelial Growth Factor; (22) PD-1/PD-L1, Programmed Death-1/Programmed Death-1 receptor; (23) HLA, Human Leukocyte Antigen; (24) RTKs, Receptor Tyrosine Kinases; (25) GPCRs, G Protein-Coupled Receptors; (26) BCR, B cell receptor; (27) TCR, T cell receptor; (28) TLR, Toll-like receptor; (29) JAK, Janus Kinase; (30) STAT, Signal Transducers and Activators of Transcription; (31) Grb2, Growth Factor Receptor-Bound Protein 2; (32) SOS, Son of Sevenless proteins; (33) RAS, RAS proteins; (34) RAF, RAF protein kinase; (35) MEK, Mitogen-activated protein kinase kinase; (36) ERK, Extracellular Signal-Regulated Kinases; (37) *RET/PTC*, *RET/PTC* oncogene; (38) PI_3_K, Phosphoinositol-3 Kinase; (39) PIP_2_:phosphatidylinositol(4,5) bisphosphate; (40) PIP_3_, Phosphatidylinositol(3,4,5) -trisphosphate; (41) AKT, Protein Kinase B; (42) mTOR, mammalian target of rapamycin; (43) Iκκα+Iκκβ+Iκκγ, IκB Kinase Complex/Iκκ Complex; (44) P50+p65/c-Rel, Nuclear Factor kappa B; (45) IκB, Inhibitor of NF-κB; (46) Ub, Ubiquitination; (47) P, Phosphorylation.

Research has established that cytokines and chemokines play a decisive role in the pathogenesis ofAITDs (1). In AITDs, infiltrating Th1 cells stimulate thyroid cells by secreting cytokines such as Interferon-γ(IFN-γ) and Tumor Necrosis Factor-α (TNF-α), which in turn induces the secretion of CXCL10 by the thyroid cells. Moreover, IFN-γis a key stimulant for the release of CXCL10 by CD4+ T cells, CD8+ T cells, and NK cells. CXCL10 interacts with the C-X-C motif chemokine receptor 3 (CXCR3) and influences the development of AITDs by activating downstream signaling pathways (26, 27). Th1 cells can also stimulate B cells to produce antibodies against thyroid antigens, such as anti-Thyroid Peroxidase Antibodies (TPOAb) and anti-Thyroglobulin Antibodies (TgAb) (**Figure 1**).

Th17 cells, a distinct subset of CD4+ T cells, are characterized by their secretion of Interleukin-17 (IL-17) and other related cytokines, including IL-21 and IL-22. Initially discovered in studies of various autoimmune diseases and inflammatory processes, Th17 cells play a pivotal role in promoting inflammatory responses.

Tregs maintain immune tolerance by suppressing the proliferation of autoreactive T cells (28). Animal studies have shown that the absence of Tregs can lead to the spontaneous development of AITDs (29). Additionally, clinical studies have demonstrated a reduction in circulating Treg cell numbers in patients with HT (30). Research indicates that the balance between Th17 and Tregs is crucial in the progression of HT, with an increased Th17/Treg ratio as HT progresses (31–33). These findings suggest that a decrease or dysfunction in Tregs may impair immune tolerance to self-antigens, thereby promoting the development of HT.

Tregs prevent thyroid damage by inhibiting autoreactive B cells that produce harmful autoantibodies targeting the thyroid, particularly those generating TPOAb and TgAb (34). Furthermore, Tregs protect thyroid tissue from immune system attacks by secreting immunosuppressive molecules such as IL-10 and Transforming Growth Factor-β(TGF-β) (35). IL-10 suppresses the activity of Th17 cells, thereby protecting the thyroid gland (**Figure 1**). Consequently, when Tregs function is compromised, the reduced secretion of IL-10 fails to effectively suppress Th17 cells, leading to increased levels of IL-17 and IL-22, which exacerbates autoimmune damage to the thyroid (36).

Recently, researchers utilized Mendelian Randomization (MR) analysis to investigate the causal relationship between 165 Treg cell markers and the risk of HT, ultimately identifying five Treg cell characteristics negatively associated with HT risk. These include Resting Treg %CD4;CD4 on resting Treg;CD28- CD8dim %CD8dim;CD25 on CD39+ resting Treg;and CD28 on activated & secreting Treg (37). Additionally, Zhao et al. demonstrated through MR analysis that an increased proportion of CD3 on CD39+ secreting Tregs and CD3 on CD4 Tregs are associated with an increased risk of HT (38). These two studies highlight the close link between Tregs and HT using genetic approaches.

Furthermore, FoxP3 and BACH2, as key regulatory proteins of the immune system, play a crucial role in maintaining the balance between Treg and Th17 cells (33), which is essential for preventing the development of HT. BACH2 functions in various immune cells, including B and T cells, promoting the generation and function of Tregs while inhibiting the excessive activation of inflammatory T cells such as Th1, Th2, and Th17. FoxP3 is predominantly expressed in Treg cells and is indispensable for their development, function, and stability (**Figure 1**). Genetic factors, epigenetic changes, abnormal post-translational modifications, and the influence of chronic inflammatory environments can lead to dysfunction of FoxP3 and BACH2. These dysfunctions may impair the suppressive function of Treg cells and abnormally activate Th17 cells (39, 40), thereby promoting the onset and progression of HT. Verginis et al. reported that TNF-α-induced semi-mature dendritic cells (DCs) can activate Treg cells by presenting thyroglobulin (Tg), thereby suppressing autoimmune responses. However, when Tregs are impaired or DCs are abnormally matured (e.g., over-activated), immune regulation is disrupted, leading to uncontrolled activation of effector T cells and potentially causing experimental thyroiditis. This finding provides a theoretical basis for developing Hashimoto’s thyroiditis treatment strategies targeting immune tolerance (41).

Programmed Death-1 (PD-1), a pivotal immune checkpoint molecule, plays an essential role in regulating T cell activity and maintaining self-tolerance. Studies have found that in the thyroid tissues of patients with AITDs, the expression of PD-1 and its ligand PD-L1 is significantly upregulated, particularly in infiltrating CD4+ and CD8+ T cells, where the increase in PD-1 expression is notably pronounced. This phenomenon suggests that the PD-1/PD-L1 signaling axis plays a crucial role in the pathogenesis of AITDs and may exert an inhibitory effect on autoimmune responses (7).

However, the specific mechanisms underlying PD-L1 expression are not fully understood: in the context of tumor cells, its expression is associated with the activation of the Mitogen-Activated Protein Kinase (MAPK) and Phosphoinositol-3 Kinase (PI_3_K) pathways (42); whereas in inflammatory conditions, interferons (IFNs) and the Signal Transducer and Activator of Transcription 1/Interferon Regulatory Factor 1 (STAT 1/IRF 1) pathway appear to be the primary inducers. It has been established that the PD-1/PD-L1 axis plays a significant role in regulating autoimmunity, but the expression patterns, functions, and relationship with disease progression of PD-L1 in AITDs require further investigation. Research by Ruiz-Riol indicates a clear increase in IFNs in AITDs (43), and IFN-γ may drive the expression of PD-L1 in Thyroid Follicular Cells (TFC) (7). PD-L1 expressed on TFC may help avoid recognition by autoreactive T cells activated in an inflammatory environment, which is crucial for maintaining peripheral tolerance. However, other studies suggest that PD-L1 expression in thyroid cells *in vivo* may be more complexly regulated than *in vitro*. This complexity involves multiple factors, including cell-cell interactions, the immune microenvironment, and the balance of signaling pathways. These factors act together to restrict PD-L1 expression. Therefore, while the PD-1/PD-L1 pathway is activated in the AITDs gland, it may not be sufficient to completely suppress disease progression (7).

As previously mentioned, in AITDs, IFNγ induces the expression of PD-L1 in TFC. Additionally, IFNγ stimulates TFC to express HLA-DR, which is crucial for maintaining peripheral immune tolerance (7). Within the context of AITDs, the expression of HLA-I on TFC is upregulated, and HLA-II are re-expressed, enabling interactions with CD4+ and CD8+ T cells (44, 45) (**Figure 1**).

In the context of HT, TFC express both HLA-I and HLA-II, PD-L1, and adhesion molecules, sending complex signals to infiltrating T cells. Rather than preventing autoimmunity, these signals tend to suppress it (44–46). In summary, the role of TFC in AITDs is multifaceted, encompassing the regulation of immune tolerance, participation in inflammatory responses, and influencing the progression of autoimmune diseases.

## 3 To explore the potential mechanisms by which HT may promote the occurrence and progression of PTC, we analyze the various differences between patients with PTC+HT and those with PTC alone

The Tumor Microenvironment (TME) encompasses the local environment where tumor cells reside and proliferate, including the tumor cells themselves as well as various cells, extracellular matrix, and cytokines that interact with the tumor cells. The TME plays a crucial role in tumorigenesis, progression, metastasis, and response to therapy.

Research has confirmed the correlation between inflammation and cancer, particularly in HT and PTC. Studies indicate that even in the absence of typical HT symptoms, PTC often exhibits significant peritumoral lymphocytic infiltration or focal lymphocytic thyroiditis, which occurs at a notably higher rate than in benign thyroid conditions (16). HT, a chronic autoimmune thyroiditis, is characterized by persistent lymphocytic infiltration and inflammatory responses within the thyroid. This sustained inflammatory state not only damages thyroid tissue but may also promote the malignant transformation of thyroid cells through various mechanisms (47). Research shows that individuals with HT are at a higher risk of developing thyroid cancer, breast cancer, lung cancer, and digestive system cancers compared to those without HT, with the risk of thyroid cancer being the most significant (48).

At the molecular level, the inflammatory environment in HT can activate various signaling pathways, including Nuclear Factor kappa-light-chain-enhancer of activated B cells (NF-κB) and the Signal Transducers and Activators of Transcription (STAT) family, which play crucial roles in tumor cell proliferation, survival, and immune evasion (49, 50). Moreover, inflammation-induced oxidative stress can lead to DNA damage and genomic instability, further promoting the progression of precancerous lesions (51).

It is noteworthy that the interaction between HT and PTC may involve a complex molecular regulatory network, including microRNAs (miRNAs), long non-coding RNAs (lncRNAs) and epigenetic modifications, among others (52). These molecular-level changes not only affect the biological behavior of thyroid cells but may also influence patients’ responses to treatment and disease prognosis.

### 3.1 HT promotes the recruitment of lymphocytes to the thyroid glands of patients with PTC

Currently, the specific mechanisms by which HT affects patients with PTC are not fully understood. Research by González-Amaro et al. confirms that HT can promote the recruitment of T lymphocytes to the thyroid, including CD4+ T cells and CD8+ T cells (53). Furthermore, Pan et al. conducted a comprehensive single-cell transcriptomic analysis of human primary PTC and found that, compared to patients with PTC alone, those with PTC+HT had an enrichment of B lymphocytes and plasma cells in tumor tissues. In PTC+HT patients, myeloid cells, such as pro-inflammatory dendritic cells (DCs), act as potential signaling hubs that continuously recruit lymphocytes into the TME. Additionally, trajectory analysis of B cells and plasma cells has shown that the enriched B lymphocytes primarily originate from adjacent HT tissues and can reshape the TME (54).

Pani et al. induced HT and PTC in mice using genetic methods to assess the impact of HT on PTC. The study revealed that mice with pre-existing thyroiditis exhibited richer immune cell infiltration compared to those with concurrent HT and PTC induction, particularly effector memory CD8+ T cells and CD19+ B cells (55). Moreover, Binnewies et al. classified the Tumor Immune Microenvironment (TIME) of PTC patients into three categories—Immune Desert (ID), Immune Exclusion (IE), and Immune Inflammation (Inf)—based on the spatial heterogeneity of CD8+ T cell distribution. The majority of PTC+HT patients exhibit the Inf phenotype, characterized by an abundance of tumor-infiltrating immune cells, including CD8+ T cells and plasma cells, indicating activation of cellular and humoral immunity (49, 56). Xing et al. examined the levels of immune cells in the serum and thyroid tissues of 680 PTC patients. The results indicated that patients with PTC+HT had a higher lymphocytic infiltration in the thyroid area compared to those with PTC alone; moreover, the infiltration level of CD8+ T cells in the thyroid tissue of PTC+HT patients was significantly higher than in PTC patients, while the levels of CD4+ T cells were lower (14). Similarly, Sulaieva et al.’s study demonstrated that PTC+HT patients had a significantly higher number of CD8+ T cells than those with PTC alone (49). Furthermore, in PTC patients, lymphocyte density is positively correlated with lower recurrence rates and improved overall survival (57). Consequently, co-existing HT affects the TME by increasing the number of CD8+ T cells within and around the tumor, thereby further activating antitumor responses.

In summary, in PTC patients with HT, the number of lymphocytes is significantly increased. This increase exerts antitumor effects through multiple mechanisms, including enhanced antigen presentation, direct tumor cell killing, cytokine secretion, and improved immune microenvironment (54, 55, 58). The synergistic action of these mechanisms significantly enhances the antitumor immune response, effectively inhibiting tumor progression and improving patient prognosis.

### 3.2 The immunosuppressive effect of Treg cells may be compromised in the context of HT

As previously discussed, Tregs are a subset of T cells with immunomodulatory functions, playing a crucial role in maintaining immune tolerance, preventing autoimmune reactions, and modulating antitumor immune responses. Their dysfunction or reduced numbers have been confirmed in AITDs (30, 41, 53, 59). Additionally, Tregs are a key component of CD4+ T cell infiltration in PTC. It has been demonstrated that Tregs participate in the M2 polarization of macrophages, which are activated by factors such as IL-4, IL-10, and IL-13 produced by Th2 cells and Tregs in an autoimmune inflammatory environment. M2 macrophages, with their anti-inflammatory properties, produce numerous growth factors that promote tumor growth and metastasis. These cytokines may aid tumors in evading immune surveillance within the tumor microenvironment, thereby facilitating tumor progression and metastasis. Moreover, M2 macrophages also promote tumor invasion by producing angiogenic factors, including Vascular Endothelial Growth Factor (VEGF) (60) (**Figure 1**). However, in the context of HT, the dysfunction of Tregs paradoxically enhances immune surveillance and thereby inhibits tumor progression.

FoxP3+ Tregs are those Tregs that express the FoxP3 protein. Sulaieva et al. evaluated the quantity and distribution of various immune cells in PTC patients using immunohistochemistry and found that the number of FoxP3+ Tregs in the intact thyroid tissue of PTC+HT patients was significantly higher than in patients with PTC alone (61). However, Sulaieva and colleagues observed that most PTC patients had very few Tregs, and although the number of Tregs in PTC+HT patients was higher, their overall quantity remained low (49). Moreover, there was no significant difference in the number of FoxP3+ Tregs between the two groups within the PTC tumor area. In patients with PTC, particularly those with PTC+HT, an increased number of Treg cells is associated with a higher risk of LNM (61).However, compared to patients with PTC alone, those with PTC+HT exhibit a lower incidence of LNM (19, 21–24).Thus, the immunosuppressive function of Treg cells may be compromised in the context of HT. Future studies could employ mouse models or *in vitro* experiments to further investigate whether Tregs in PTC patients with HT exhibit functional defects or activation barriers.

Overall, the impact of HT on the PTC immune microenvironment is complex and multifaceted. Future research should focus on elucidating the specific mechanisms by which Tregs function in the coexistence of PTC and HT, as well as their roles in immune surveillance and immune evasion.

### 3.3 IFN-γ can promote cell proliferation and tumor transformation through the CXCR3A-CXCL10 axis

CXCR3 is a chemokine receptor belonging to the CXC chemokine receptor subgroup. It is predominantly expressed on the surface of activated T cells, B cells, and NK cells, inducing targeted migration and immune responses by binding to specific receptors on target cell membranes, and plays a significant role in infections, autoimmune diseases, and tumor immunity. CXCR3 and its ligands CXCL9, CXCL10, and CXCL11 are closely associated with the occurrence and progression of various tumors (26). CXCR3 has two splice variants, CXCR3A and CXCR3B, which promote cell proliferation and induce apoptosis, respectively. CXCR3 ligands, such as CXCL4, CXCL9, CXCL10, and CXCL11, may elicit antagonistic responses. Notably, CXCR3A signaling primarily promotes cell proliferation, angiogenesis, and inhibits apoptosis through CXCL10 and CXCL11 (62). It has been demonstrated that in human breast, ovarian, renal, and prostate cancer tissues, the expression of CXCR3A is upregulated, while the expression of CXCR3B is significantly reduced (63–66).

As previously mentioned, Th1 cells infiltrating AITDs secrete cytokines such as IFN-γ and TNF-α, with IFN-γ inducing the secretion of CXCR3 ligands by both normal and PTC thyroid cells (67, 68) (**Figure 1**). Urra et al. revealed that the levels of both CXCR3 splice variants are upregulated in both benign and malignant thyroid tumors, with CXCR3A being the predominant isoform. Further cell transfection and MTT proliferation assays demonstrated that CXCR3A significantly promotes cell proliferation in Nthy-ori-3-1 cells via stimulation by its ligands CXCL10 and CXCL11. However, CXCL10 is upregulated only in patients with PTC+HT (69). Overall, inflammation enhances signaling through the CXCR3A-CXCL10 axis, activates the PI_3_K/AKT and MAPK pathways, thereby promoting cell proliferation, inhibiting apoptosis, and modulating immune cell infiltration. These effects collectively drive tumor transformation and cancer progression (65, 70).

### 3.4 IL-2 expression is upregulated in tissues from patients with PTC+HT, promoting the expression of MHC-I molecules

As previously discussed, the PD-1/PD-L1 co-stimulatory signal negatively regulates T cell immunity, facilitating tumor evasion of immune surveillance. The expression of MHC-I is crucial for antitumor CD8+ T cell immunity. Another mechanism by which tumors evade immune destruction is the low expression of MHC-I on tumor cells, which prevents effective presentation of antigenic determinants to activate CD8+ T cell immunity (71). The absence of MHC-I expression is one of the common characteristics in tumor tissues.

IL-2 is a pleiotropic cytokine that promotes the proliferation of activated T cells. Hu et al. first analyzed the expression of IL-2 and MHC-I in thyroid tissues from PTC and PTC+HT patients using qRT-PCR and Western blot, finding that IL-2 expression was upregulated in thyroid tissues from PTC+HT patients compared to those with PTC alone, and it positively correlated with MHC-I expression. Subsequently, the authors demonstrated through *in vitro* co-culture that PTC cells treated with IL-2 significantly enhanced the proliferation and cytokine production of effector CD8+ T cells, but reduced PD-1 expression in activated CD8+ T cells and PD-L1 expression in PTC cells. In summary, the study suggests that T cells infiltrating the thyroid tissues of PTC+HT patients secrete IL-2, which directly upregulates MHC-I expression, enhances immune cell infiltration, and promotes T cell activation. These mechanisms collectively enhance the immunogenicity of PTC cells, overcome immune evasion, and thereby limit tumor progression (72). These findings provide a plausible explanation for the better prognosis typically observed in patients with both HT and PTC. Currently, recombinant IL-2 has been used to treat autoimmune diseases and malignancies (73–75). However, its potential application in PTC treatment warrants further investigation.

### 3.5 In HT, inflammatory cells can activate the NF-κB pathway, thereby promoting tumorigenesis

NF-κB is a pivotal transcription factor that plays a significant role in inflammation-mediated tumor progression. Its activation can enhance tumor cell proliferation, survival, and invasion. CD25, the α-chain of the interleukin-2 receptor(IL-2Rα), is expressed in Treg cells and B cells and is a component of the NF-κB pathway; increased expression may indicate pathway activation (**Figure 1**). Researchers assessed the expression of CD25 and phosphorylated NF-κB (p-NF-κB) in the cytoplasm and nucleus of PTC and HT patients using immunohistochemical staining. The results suggest that in HT, the presence of inflammatory cells may activate the NF-κB pathway by releasing cytokines and chemokines, thereby promoting tumorigenesis and development. In PTC, increased CD25 expression and aberrant NF-κB pathway activation can lead to increased cell proliferation and anti-apoptotic protein expression, suppress immune responses, and promote tumor invasion and metastasis. Moreover, immunopositivity for p-NF-κB correlates with a multifocal growth pattern (50). Curcumin has been shown to exert antitumor effects on PTC cells through multiple mechanisms, including inhibiting NF-κB activity, inducing cell cycle arrest, promoting apoptosis, and modulating miRNA expression. Although laboratory studies have revealed its potential antitumor effects on PTC, curcumin has not yet been applied in clinical treatment of PTC patients due to its low bioavailability, rapid *in vivo* metabolism, and poor targeting (76). Future research should focus on improving its tumor targeting and exploring combination therapies to fully realize the potential of curcumin in PTC treatment.

### 3.6 Inflammation triggers *RET/PTC* gene rearrangements, activating the immune system and thereby promoting the transformation of thyroid cells into cancer

The receptor tyrosine kinase encoded by the *RET* gene activates the MAPK signaling pathway through ligand binding under normal physiological conditions, thereby regulating cell differentiation and proliferation (77). The presence of large amounts of free radicals and oxidative stress, as well as high levels of cytokines and chemokines in the inflammatory environment, provides favorable conditions for the occurrence of *RET/PTC* rearrangements (16). Rhoden and colleagues used three detection methods—fluorescence *in situ* hybridization (FISH), quantitative real-time RT-PCR, and laser capture microdissection (LCM)-RT-PCR—to study 43 samples from 31 HT patients without tumors. The results showed that the incidence of *RET/PTC* rearrangements in HT patients without PTC was approximately 62%, but the rearrangement levels were low, with mRNA levels equivalent to fewer than one *RET/PTC1*-positive cell per 10,000 negative cells. Moreover, these low-level *RET/PTC* rearrangements did not lead to imbalances in *RET* gene expression (78).

*RET/PTC* rearrangements are considered a key mutational event in thyroid cell carcinogenesis. Even in the absence of HT, factors such as radiation exposure, chemical carcinogens, and genetic mutations can induce genomic instability in thyroid cells, leading to *RET/PTC* rearrangements (16). The incidence of *RET/PTC* rearrangements in PTC varies across studies, with an overall range of 20% to 70% (77). *RET/PTC* rearrangements promote tumorigenesis by activating the MAPK, PI_3_K/AKT, and NF-κB signaling pathways and are associated with aggressive phenotypes, such as larger tumor size and late-stage diagnosis (77, 79–81). *In vitro* experiments have demonstrated that thyroid cells with *RET/PTC* rearrangements exhibit significant malignant features, including accelerated cell proliferation, enhanced anti-apoptotic capacity, and increased invasiveness (82). Moreover, Nikiforova et al. detected *RET/PTC* rearrangements in PTC patients without HT (83). These studies further confirm the oncogenic potential of *RET/PTC* rearrangements in the absence of HT.

In contrast, in the context of HT combined with PTC, cells with *RET/PTC* rearrangements can activate an autonomous pro-inflammatory transcriptional program through a series of cascading reactions. This process not only induces the secretion of pro-inflammatory cytokines and chemokines (such as CXCL10 and its receptor CXCR3) but also attracts more inflammatory cells, forming a positive feedback loop that further promotes PTC progression (16). Russell et al. demonstrated through a *RET/PTC3* transgenic mouse model that *RET/PTC3* activates the NF-κB signaling pathway, induces pro-inflammatory cytokine secretion, and promotes inflammatory cell infiltration, thereby driving PTC progression (81). Additionally, Powell et al. observed in the transgenic mouse model that the expression of *RET/PTC* not only induces the development of PTC but is also associated with the occurrence of chronic thyroiditis (84). Moreover, cytokines and chemokines released from the inflammatory tumor stroma can maintain the survival of thyroid cells with *RET/PTC* rearrangements, rendering them resistant to oncogene-induced apoptosis. Therefore, in patients with HT and PTC, the incidence of *RET/PTC* rearrangements is higher, and its expression levels are significantly greater than in patients with HT alone (16). Numerous molecular genetic studies on the impact of *RET/PTC* oncogene activation on the immune system support a potential pathogenic link between PTC and HT (16, 85–87).

In summary, the presence of HT significantly increases the likelihood of *RET/PTC* rearrangements. These rearrangements promote the development and progression of PTC by activating the MAPK, PI_3_K/AKT, and NF-κB signaling pathways. Given the critical role of *RET/PTC* rearrangements in PTC, inhibitors targeting downstream signaling pathways, as well as gene and immunotherapy strategies, hold promise for providing more precise and effective treatment options for PTC patients. However, whether *RET/PTC* rearrangements can serve as a prognostic marker remains to be further investigated.

### 3.7 Thyroid-stimulating hormone stimulation of thyroid cell proliferation may promote the development of PTC

Thyroid-stimulating hormone (TSH) is a heterodimeric glycoprotein hormone synthesized by thyrotrope cells in the pituitary gland. Studies have found that high levels of TSH are associated with PTC in the context of HT. Most HT patients present with hypothyroidism, which is characterized by chronically elevated TSH levels. This chronically elevated TSH stimulates the proliferation of thyroid follicular epithelial cells through its G-protein-coupled receptor (TSHR) on the thyroid cell surface. The primary biological activity of TSH is mediated by increasing intracellular cAMP levels. Increased cAMP levels activate multiple downstream signaling pathways, including cAMP/PKA, PI_3_K/AKT, MAPK, and PLC/PKC (88). These pathways play crucial roles in thyroid cell proliferation, survival, and dedifferentiation, thereby promoting the development and progression of PTC. Additionally, TSH-induced signaling pathways are involved in cell cycle regulation, gene expression regulation, and the secretion of inflammatory factors. The synergistic actions of these pathways not only promote abnormal thyroid cell proliferation but also potentially drive PTC development and progression by inducing immune evasion and altering the inflammatory microenvironment (6, 89–91). Multiple therapeutic strategies targeting TSH-induced PTC development can block TSH action and its signaling pathways from different angles. These strategies include inhibiting TSH secretion, blocking TSHR, suppressing downstream signaling pathways, and modulating thyroid function. Future research should further explore the clinical potential of these mechanisms and develop more effective therapeutic approaches to improve the prognosis of PTC patients.

### 3.8 STAT6 expression is higher in patients with PTC+HT compared to those with PTC alone and is correlated with the extent of lymphocytic infiltration

STAT6 is a crucial transcription factor involved in various cellular signaling pathways, particularly in immune responses and inflammatory processes. It mediates signals from cytokines such as IL-4 and IL-13, promoting the polarization of M2 macrophages (92). Activation of STAT6 may influence the inflammatory environment and tumor development in HT patients, and in PTC patients, it may promote tumor progression and metastasis by affecting immune cell polarization and angiogenesis (**Figure 1**). Sulaieva et al. found through immunohistochemical analysis that STAT6 expression levels were significantly higher in PTC patients with HT than in those with PTC alone and were positively correlated with lymphocyte infiltration density. The study results suggest that when HT and PTC coexist, increased STAT6 expression promotes the polarization of M2 macrophages and regulates the tumor immune microenvironment, potentially enhancing tumor immune evasion (49). Thus, STAT6 could be a potential therapeutic target for modulating immune responses and inhibiting tumor growth in PTC and HT patients. Future research should further explore the detailed mechanisms of STAT6 in PTC and HT, as well as its impact on the tumor’s immune microenvironment and therapeutic responses. Although direct therapeutic strategies targeting STAT6 in PTC are not yet fully mature, its key role in the tumor immune microenvironment offers broad prospects for future therapeutic exploration. With the advancement of in-depth basic research, systematic preclinical experiments, and rigorous clinical trials, it is hoped that innovative STAT6-based therapeutic approaches can be developed to significantly improve the prognosis of PTC patients.

### 3.9 In comparison to patients with PTC alone, those with PTC+HT show increased expression of E-cadherin and TGF-β, and decreased expression of N-cadherin and ICAM-1 in thyroid tissue

Cadherins are a family of transmembrane proteins that require calcium ions to function, playing a crucial role in regulating cell adhesion and maintaining tissue structure. E-cadherin is predominantly expressed in epithelial tissues, while N-cadherin is primarily found in neurons. In cancer development, including thyroid cancer, E-cadherin expression is inversely correlated with tumor invasiveness and metastatic potential (93, 94). In contrast, in PTC, N-cadherin not only promotes tumor growth by facilitating the Epithelial-Mesenchymal Transition (EMT) process but is also closely associated with the activation of the MAPK/ERK, PI_3_K/AKT, and p16/Rb signaling pathways. The activation of these pathways may enhance tumor cell proliferation, survival, and invasion, thereby promoting the progression of PTC (95). Kim et al. found that, compared to normal tissue, the expression of E-cadherin and N-cadherin is increased in thyroid tissues from PTC and PTC+HT patients. However, compared to PTC patients, PTC+HT patients exhibit higher E-cadherin expression and lower N-cadherin expression in thyroid tissues (96).

TGF-β is a family of cytokines with three subtypes. Under physiological conditions, TGF-β expressed in thyroid tissue regulates growth and function by phosphorylating Smad proteins (97). Studies have shown that in patients with PTC+HT, TGF-β expression in thyroid tissue is higher than in patients with PTC alone. Additionally, plasma TGF-β levels in these patients are higher than in those with PTC alone but lower than in healthy controls (96). Consequently, TGF-β plays a significant role in the development of PTC.

ICAM-1, a cell surface receptor belonging to the immunoglobulin superfamily of adhesion molecules, is expressed in a variety of cells. ICAM-1 expression has been shown to be upregulated in patients with PTC and correlates with tumor aggressiveness (98). Studies confirm that in patients with PTC+HT, ICAM-1 expression is lower than in those with PTC alone (96).

In summary, Kim et al. demonstrated through immunohistochemical analysis of 34 thyroid tissue samples that the presence of HT may reduce the tumor invasiveness and metastatic potential of PTC by upregulating the expression of E-cadherin and TGF-β, and downregulating the expression of N-cadherin and ICAM-1 (96). This discovery offers novel insights into the molecular mechanisms underlying the co-occurrence of HT and PTC. Future research should delve into these mechanisms with the aim of developing innovative therapeutic strategies.

### 3.10 Compared to patients with PTC alone, those with PTC + HT exhibit higher expression of *DMBT1*

The Deleted in Malignant Brain Tumors 1 (*DMBT1*) gene, located on the long arm of chromosome 10, is composed of highly homologous repetitive exons and intron sequences. Studies suggest that the *DMBT1* gene has potential tumor-suppressing functions, with its expression closely associated with immune defense, cellular polarization, differentiation, and regeneration (99). Notably, the expression levels of *DMBT1* in normal tissues are significantly higher than in various cancerous or diseased tissues, including autoimmune diseases, breast cancer, colon cancer, and prostate cancer (100–102).

It has been established that high expression of the *DMBT1* gene correlates with lower clinical staging, reduced risk of lymph node metastasis, and smaller tumor diameter (103), suggesting that *DMBT1* may play a role in inhibiting the progression of PTC. The study by Gan et al. further supports this notion. They analyzed PTC data from the TCGA database and validated their findings with tissue samples collected from the First People’s Hospital of Guangzhou to investigate the impact of the *DMBT1* gene on PTC+HT. The results showed that *DMBT1* expression was significantly higher in the PTC+HT group compared to the PTC group; high *DMBT1* expression was associated with lower risks of clinical staging, lymph node metastasis, and tumor size; the overall immune activity in the high *DMBT1* expression group was greater than in the low expression group; important *HLA* genes were highly expressed in the high *DMBT1* expression group; and there were significant differences in key immune checkpoint genes, including *CTLA-4* and *IDO1*, between high and low *DMBT1* expression levels (3). In summary, high expression of *DMBT1* in PTC+HT patients may be a potential tumor suppressor factor, and high *DMBT1* expression may improve PTC prognosis through immune-related pathways. However, the specific mechanisms behind the high expression of the *DMBT1* gene in PTC+HT patients remain unclear and may be related to the immune characteristics of HT and the role of *DMBT1* in immune regulation, warranting further investigation.

### 3.11 In HT, inflammation-induced oxidative stress can promote the development of precancerous lesions

Studies have shown that reactive oxygen species (ROS) are upregulated in HT, directly attack DNA, cause DNA damage, and activate DNA repair mechanisms, leading to dysregulation of DNA repair-related genes. Subhi et al. analyzed the gene expression profiles of HT and PTC coexistence using microarrays and found that lymphocytic infiltration is closely associated with chronic ROS exposure. Additionally, studies have shown that in the context of HT and PTC coexistence, genes related to ROS metabolism, DNA damage, and repair are significantly upregulated in PTC cells, and their abnormal expression is closely linked to the aggressive features of PTC. This chronic oxidative stress and the cumulative effects of DNA damage provide a favorable genetic background for the development of PTC. These findings reveal the potential link between inflammation-induced oxidative stress and genetic variations or chromosomal abnormalities in PTC cells (51, 104). Therefore, antioxidant therapy and regulation of DNA damage repair can alleviate oxidative stress and DNA damage, thereby improving patient prognosis.

## 4 Summary and outlook

In summary, HT is closely associated with PTC. PTC with HT exhibits a distinct immune microenvironment and molecular profile, involving the recruitment and activation of immune cells, as well as multiple signaling pathways related to tumor transformation and progression. Specifically, the progression of PTC in the context of HT is influenced by a complex interplay of mechanisms that both promote tumor development and inhibit tumor progression.

On the one hand, the chronic inflammatory environment in HT promotes the development and progression of PTC through multiple mechanisms. Increased IFN-γ secretion activates the CXCR3A-CXCL10 axis, which in turn activates the PI_3_K/AKT and MAPK pathways, promoting cell proliferation, inhibiting apoptosis, and regulating immune cell infiltration. Cytokines and chemokines released by inflammatory cells activate the NF-κB pathway, further driving tumor development. Moreover, HT significantly increases the incidence of *RET/PTC* rearrangements, which further promote PTC development by activating the MAPK, PI_3_K/AKT, and NF-κB pathways. Concurrently, elevated TSH levels stimulate the proliferation of thyroid follicular epithelial cells and activate signaling pathways such as cAMP/PKA, PI_3_K/AKT, MAPK, and PLC/PKC by increasing intracellular cAMP levels, thereby driving PTC development. Additionally, elevated ROS levels in HT directly damage DNA, activate DNA repair mechanisms, and cause dysregulation of related genes, thereby promoting PTC development.

On the other hand, HT also inhibits PTC progression through multiple mechanisms. HT promotes the recruitment of lymphocytes to the thyroid, reshapes the tumor microenvironment, and enhances anti-tumor immune responses. Increased IL-2 secretion directly upregulates MHC-I expression, enhances immune cell infiltration and T cell activation, increases the immunogenicity of PTC cells, and overcomes immune evasion, thereby limiting tumor progression. Moreover, dysfunction of Tregs further enhances immune surveillance and inhibits tumor progression. Upregulation of E-cadherin and TGF-β, and downregulation of N-cadherin and ICAM-1, inhibit tumor growth by suppressing EMT and reduce the invasiveness and metastatic potential of PTC by activating the MAPK, PI_3_K/AKT, and p16/Rb pathways. Concurrently, increased expression of the *DMBT1* gene, which is associated with immune defense, cell polarization, differentiation, and regeneration, exerts potential anti-tumor effects.

Therefore, although HT may promote the development of PTC, it also inhibits tumor progression by enhancing immune surveillance and modulating the tumor microenvironment. This paradoxical effect results in lower tumor invasiveness and better prognosis in PTC patients with HT.

The findings underscore the importance of delving into the interactions between HT and PTC, which is crucial for uncovering the complex pathological mechanisms between the two and provides a solid scientific foundation for developing innovative treatment strategies. Future research should focus on elucidating the precise molecular mechanisms by which HT influences signaling pathways and promotes the development of PTC, enabling the formulation of effective therapeutic strategies to prevent the progression of HT to PTC. Additionally, investigating the role of immune cells in the tumor microenvironment of PTC patients with HT may offer new directions for the development of novel immunotherapeutic strategies targeting HT-related PTC. Considering the characteristics of PTC patients with HT, such as multifocality, smaller tumor size, and lower risk of lymph node metastasis, a more nuanced and personalized consideration of thyroidectomy and lymph node dissection strategies is warranted. In light of the risks associated with postoperative TSH suppression therapy in PTC patients, such as increased cardiovascular and fracture risks, it is essential to further investigate whether PTC patients with HT, under otherwise identical conditions, could achieve similar tumor recurrence suppression effects with relatively higher TSH suppression target values compared to those without HT.
